# Supplementary material for: MetaRibo-Seq measures translation in microbiomes
Source: Nat Commun. 2020 Jun 29;11:3268. doi: 10.1038/s41467-020-17081-z (PMC7324362; doi:10.1038/s41467-020-17081-z)
Supplement: Supplementary file 10 — Supplementary Data 7 [file 41467_2020_17081_MOESM10_ESM.zip › File2/Confidence_High_Taxonomy/29703_out.krona.html]

Javascript must be enabled to view this page.

members
magnitude
magnitudeUnassigned
count
unassigned
taxon
rank

29703\_out

67

2
67
superkingdom

phylum
67
976

200643
67
class

171549
67
order

2005525
60
family

60
genus

SRS015369\_contig\_number\_3126SRS017521\_contig\_number\_29784SRS018575\_contig\_number\_2681SRS024625\_contig\_number\_1757SRS048164\_contig\_number\_26996SRS050974\_contig\_number\_55SRS056519\_contig\_number\_15320SRS077294\_contig\_number\_3270SRS078176\_contig\_number\_1057
375288
9

2

SRS013638\_contig\_number\_10977SRS075878\_contig\_number\_21720
species
2293123

18

SRS011084\_contig\_number\_11714SRS012969\_contig\_number\_22789SRS015217\_contig\_number\_30288SRS016267\_contig\_number\_11622SRS019496\_contig\_number\_4320SRS050925\_contig\_number\_20788SRS051610\_contig\_number\_9433SRS051882\_contig\_number\_11233SRS062701\_contig\_number\_1263SRS077849\_contig\_number\_11754SRS1041091\_contig\_number\_20109SRS1041129\_contig\_number\_12062SRS1041142\_contig\_number\_7188SRS1041145\_contig\_number\_29528SRS104693\_contig\_number\_1640SRS1055038\_contig\_number\_6058SRS143876\_contig\_number\_26114SRS148511\_contig\_number\_5628
species
1262912

823
29
species

SRS011134\_contig\_number\_19413SRS014287\_contig\_number\_12721SRS014313\_contig\_number\_2026SRS014459\_contig\_number\_1751SRS015065\_contig\_number\_16736SRS015431\_contig\_number\_32441SRS015890\_contig\_number\_14013SRS018656\_contig\_number\_8607SRS018984\_contig\_number\_5661SRS019068\_contig\_number\_52094SRS019397\_contig\_number\_3260SRS019910\_contig\_number\_24757SRS022609\_contig\_number\_15453SRS045244\_contig\_number\_3605SRS046369\_contig\_number\_1683SRS048060\_contig\_number\_8178SRS048870\_contig\_number\_7300SRS053398\_contig\_number\_23210SRS054590\_contig\_number\_59SRS058145\_contig\_number\_3824SRS058723\_contig\_number\_2848SRS064557\_contig\_number\_3310SRS078242\_contig\_number\_11561SRS098061\_contig\_number\_2899SRS1041090\_contig\_number\_5646SRS142712\_contig\_number\_5160SRS143085\_contig\_number\_589SRS893252\_contig\_number\_4786SRS971275\_contig\_number\_7890

328812
1
species

SRS011239\_contig\_number\_2589

species
1

SRS098717\_contig\_number\_17120
46503

815
family
7

816
7
genus

species
1

SRS063370\_contig\_number\_2265
338188

species
1

SRS023526\_contig\_number\_24072
46506

371601

SRS019787\_contig\_number\_8608
1
species

1

SRS077335\_contig\_number\_4660
species
871324

2
species

SRS019968\_contig\_number\_12765SRS1055049\_contig\_number\_11949
821

1

SRS020622\_contig\_number\_1109
species
674529
